# Determination and Correlation Analysis of Mineral Elements in Soil, Forage, and Biological Samples (Hair and Serum) from Yaks in Ganzi Prefecture, China

**DOI:** 10.3390/vetsci13060573

**Published:** 2026-06-10

**Authors:** Zhidi Xu, Yao Pan, Wei Tian, Qingting Yu, Qiang Li, Xing Hu, Zhicai Zuo, Yue Xie, Xiaoping Ma, Lan Lan, Hongrui Guo

**Affiliations:** 1 College of Veterinary Medicine, Sichuan Agricultural University, Wenjiang, Chengdu 611130, China; 17835424879@163.com (Z.X.); czyhzzc@126.com (Z.Z.); xyue@sicau.edu.cn (Y.X.); mxp886@sicau.edu.cn (X.M.); 2 Animal Husbandry Science Institute of Ganzi Tibetan Autonomous Prefecture, Kangding 626000, China; panyao199302@163.com (Y.P.); 18892893381@163.com (W.T.); yuqingtingxukesuo@163.com (Q.Y.); lq18782276368@163.com (Q.L.); l8283642146@163.com (X.H.)

**Keywords:** yak calves, hair, serum, forage, soil, trace elements, altitude, Ganzi prefecture

## Abstract

Mineral imbalances in livestock can severely impair growth, immunity, and productivity. Yaks inhabiting the Qinghai–Tibet Plateau face unique environmental challenges, including altitude-induced nutritional deficiencies. This study investigated the mineral status of healthy yak calves and its correlation with altitude across six regions in Ganzi Prefecture at elevations of 3200–4200 m, as well as the interrelationships among mineral elements in the “soil–forage–animal” ecosystem. A total of 42 hair samples and 42 serum samples were collected from yak calves across the six regions, and 49 forage samples and 49 soil samples were collected from seven regions. The contents of 11 mineral elements (Na, K, Ca, Mg, S, Cu, Fe, Mn, Zn, Co, and Se) were determined in all samples. The results revealed widespread deficiencies. The primary deficiencies were as follows: Na in hair was significantly lower than reference values, with deficiency rates ranging from 2% to 48.1%; Co was deficient to varying degrees in all samples, with deficiency rates ranging from 54.0% to 89.5%; Se was also commonly deficient, with rates between 32.7% and 74.7%. In serum, Mg, S, and Cu were generally deficient, with deficiency rates as high as 39.3%, 41.3%, and 47.2%, respectively. Serum Se deficiency occurred frequently at high altitudes, particularly in Seda and Shiqu counties. Additionally, significant correlations were observed: negative correlation between forage Zn and hair Zn in Luhuo, positive correlation between forage S and hair S in Ganzi, negative correlation between forage Se and serum Se in Yajiang, positive correlation between soil Se and forage Se as well as between soil Na and hair Na in Seda, positive correlation between forage Co and serum Co in Shiqu, and positive correlations between soil Se and serum Se and between forage Co and hair Co in Jiulong. These findings indicate that multiple mineral deficiencies are prevalent in yak calves, are closely associated with altitude, and may be influenced by soil and forage mineral composition as well as feeding practices. This study emphasizes the urgent need to develop targeted mineral supplementation strategies to support yak health and growth.

## Simple Summary

Yaks are an indispensable livestock species on the Qinghai–Tibetan Plateau, particularly in Ganzi Prefecture, providing local herders with meat, milk, and economic stability. However, the high-altitude environment presents specific nutritional deficiencies, particularly regarding mineral elements. In this study, we measured the concentrations of 11 mineral elements in hair and serum samples from healthy yak calves, as well as in forage and soil samples collected from their habitats. The results indicated that deficiencies in elements such as selenium, cobalt, magnesium, and sodium were quite common, and the concentrations of some elements were closely correlated with altitude. Furthermore, correlation analysis of mineral element contents among soil, forage, hair, and serum revealed complex interrelationships. These correlations varied across the six regions, primarily attributable to regional differences in soil and forage mineral levels, livestock activity, and interactions among elements. These findings highlight the necessity of targeted mineral supplementation to improve yak health and productivity. Our results provide practical guidance for yak breeders and veterinary professionals working in high-altitude environments.

## 1. Introduction

Yaks (*Bos grunniens*) represent the cornerstone of livestock husbandry in Ganzi Prefecture, Sichuan Province, and are an endemic species of the Qinghai–Tibetan Plateau that has long adapted to extreme environments, serving as indispensable resources for local production and livelihoods [1,2]. Renowned for their remarkable physiological resilience, yaks thrive in harsh environments characterized by hypoxia, low temperatures, and intense ultraviolet radiation. However, this adaptation is critically dependent on the internal homeostasis of various essential mineral elements. Deficiencies in these elements impair physiological function and productivity, compromising the efficient development of the regional economy [3]. Ensuring adequate intake of essential nutrients remains challenging due to the extensive grazing-based livestock systems predominant in the Ganzi region [4,5]. Furthermore, seasonal fluctuations in forage availability and regional soil mineral compositions contribute to inconsistent patterns of nutritional deficiency across different pastoral zones. Accurate nutritional assessment necessitates localized sampling and targeted analyses to inform region-specific supplementation strategies. To date, limited studies have systematically evaluated mineral element status in yak calves across varying altitude gradients, and investigations exploring mineral element levels and their interrelationships within the soil–forage–livestock ecosystem in Ganzi Prefecture remain scarce. This study addresses these gaps by providing foundational data to guide targeted nutritional interventions.

Serum analysis is commonly employed to assess mineral status in livestock, as it reflects the current physiological state and enables dynamic monitoring of nutritional or disease responses. Although less frequently utilized, hair analysis offers complementary value by reflecting long-term mineral deposition and metabolic trends [6]. Hair is easily collected, remains stable during storage, and is suitable for retrospective mineral evaluation. Certain elements, such as zinc and selenium, accumulate at higher concentrations in hair, rendering changes more readily detectable [7]. Furthermore, studies have demonstrated correlations between mineral levels in hair and serum concentrations of specific elements. Therefore, the combined application of serum and hair analyses provides a more comprehensive assessment of both short-term dynamics and long-term mineral status, particularly within the context of environmental influences such as altitude gradients [8].

The maintenance of mineral homeostasis, encompassing both macroelements and trace elements, is essential for the health and productivity of livestock [9]. Soil pH is a core factor influencing forage mineral element bioavailability. For instance, under the specific soil conditions of alpine meadows on the Qinghai–Tibetan Plateau, soil pH may be associated with carbon cycling and nutrient mineralization processes [10]. Studies have demonstrated that soil pH becomes a key driving factor affecting nutrient release during the later stages of litter decomposition. In alpine meadows, compared with moist soils (e.g., riparian zones), the entire plant community on sunny slopes exhibits the highest forage value [11]. Studies have demonstrated that the concentrations of macroelements and trace elements in forage directly influence mineral element concentrations in yak serum [12]. Mineral deficiencies in forage exert profound effects on yak health through the food chain. In the Tianjun region of Qinghai, elevated molybdenum content in forage led to an imbalanced molybdenum-to-copper ratio, inducing secondary copper deficiency in yaks, which manifested as emaciation, unsteady gait, and “swayback” symptoms [13]. Similarly, insufficient phosphorus content in forage caused “stiff limb disease” in yaks, characterized by emaciation, lameness, and rigid gait [14]. Numerous studies have confirmed that mineral element concentrations in soil, forage, and livestock exhibit significant regional variations attributable to geological and environmental heterogeneity. Moreover, these elements demonstrate varying degrees of correlation among the three compartments [15]. The contents and distribution of mineral elements in soils are primarily dependent on the parent materials of naturally developed soils, as well as on vegetation growth and environmental pollution [16,17]. In contrast, mineral element concentrations in forage are closely associated with plant species/cultivar, growth and physiological stages, and seasonal and climatic conditions [18].

This study aimed to investigate the mineral element status of healthy yak calves in Ganzi Prefecture, as well as the mineral element contents in grazing soil and forage. By analyzing eleven key mineral elements in yak hair, serum, soil, and forage samples, this research elucidated deficiency patterns and spatial distribution characteristics along different altitude gradients, and explored the mineral element levels and their interrelationships within the “soil–forage–animal” ecosystem. The findings provide a scientific basis for guiding targeted mineral supplementation and ecological management strategies, thereby supporting yak health and promoting sustainable development of animal husbandry in high-altitude pastoral areas.

## 2. Materials and Methods

### 2.1. Experimental Animals

**Figure 1:**
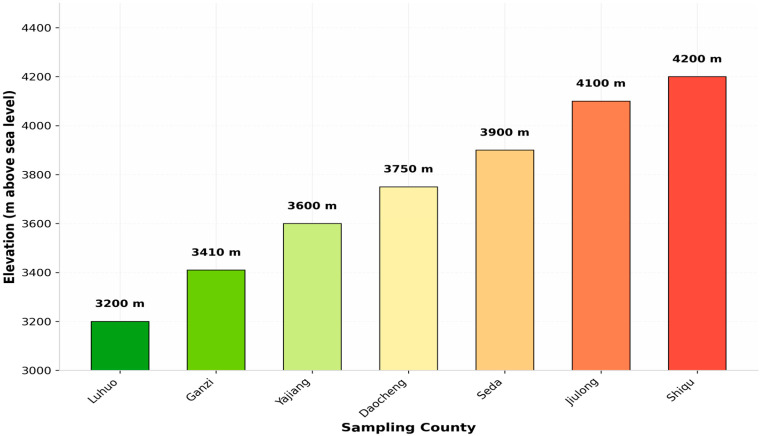
Elevation distribution of seven sampling counties in Ganzi prefecture (m above sea level).

This study was conducted across seven high-altitude pastoral regions in Ganzi Prefecture, Sichuan Province, China. Sampling was conducted in August 2025 at seven locations: Daocheng County (3750 m), Luhuo County (3200 m), Ganzi County (3410 m), Yajiang County (3600 m), Seda County (3900 m), Shiqu County (4200 m), and Jiulong County (4100 m), representing typical altitudinal gradients of the yak plateau ecosystem (in Figure 1). Except for Daocheng County, a total of 42 clinically healthy yaks aged 1–2 years were randomly selected from the remaining six regions, with seven yaks chosen from each region. All animals were assessed by local veterinary personnel and exhibited no overt signs of disease or malnutrition at the time of sampling. The animal use protocol listed below has been reviewed and approved by the Sichuan Agricultural University Animal Ethical and Welfare Committee (Approval No. 20260275).

### 2.2. Hair Sample Collection

Hair sample collection procedures followed standard veterinary methods as described by Sizova [6] and Lim [7] ensuring consistency with established sampling protocols. Approximately 10 g of hair was collected from the dorsal neck region of each animal using clean stainless steel scissors. To avoid external contamination, the sampling area was cleaned prior to collection. The samples were sealed in sterile polyethylene bags and stored at −20 °C until analysis.

### 2.3. Serum Sample Collection

Serum sample collection procedures were consistent with the sampling protocols described in the literature cited in Section 2.2. Jugular blood was collected aseptically using vacuum tubes without anticoagulant. Samples were transported on ice and centrifuged at 3000 rpm for 10 min within 6 h of collection. The separated serum was aliquoted and stored at −80 °C until mineral analysis.

### 2.4. Forage Sample Collection

Forage sampling was conducted in accordance with the FAIR data principles [19]. Sampling sites were selected to represent diverse geographical features, elevations, soil types, and grassland utilization patterns, specifically targeting large-scale grazing areas within each region. A “tracking grazing sampling method” was employed, whereby sampling points were established by following grazing herds within their activity areas to identify three representative sites. Edible forage species from various categories were collected at each site (in Table 1). Seven forage samples were collected per region, yielding a total of 49 samples across seven regions. At each sampling point, above-ground portions of various edible forage species (typically 2–3 cm above ground level) were collected within a fixed quadrat area of 4 m^2^, thoroughly mixed, and combined to form a single composite sample [20]. Stainless steel scissors or sickles were used for collection to prevent metal contamination. Fresh forage samples were immediately placed in clean polyethylene self-sealing bags or cloth bags and stored in an insulated container with ice packs at 4 °C.

### 2.5. Soil Sample Collection

Concurrent with forage sampling, corresponding soil samples were collected using a grid-based sampling method. The sampling area was divided into square grids of 10 m × 10 m, and soil samples were collected at the center, nodes, or intersections of each grid. Stainless steel shovels or bamboo spatulas were used as sampling tools to avoid contamination. Samples were collected from a depth of 0–20 cm and immediately placed in clean cloth bags or polytetrafluoroethylene (PTFE) plastic bags. Seven soil samples were collected per region (1 kg per sample), yielding a total of 49 samples across seven regions [21,22,23]. All samples were stored in a cool, ventilated, and dry environment.

### 2.6. Mineral Element Analysis

An air-dried soil sample (0.1000 g) that passed through a 0.149 mm nylon sieve was weighed into a polytetrafluoroethylene (PTFE) crucible. The sample was moistened with a small amount of water, followed by the addition of 5 mL of Hydrofluoric Acid and 2 mL of HClO_4_. The mixture was heated on a hot plate until the white fumes were completely exhausted, and then allowed to cool. Subsequently, 5 mL of 1:1 HCl solution was added, and the residue was dissolved by heating. After cooling, the solution was transferred to a 50 mL volumetric flask and diluted to volume with water. An aliquot of 5.00 mL of the above solution was transferred into a 50 mL volumetric flask and diluted to volume with water.

Each hair, serum and forage sample was homogenized, and an appropriate amount was weighed into a microwave digestion vessel. Subsequently, 6 mL of analytical-grade nitric acid (HNO_3_) was added, and samples were left to stand overnight for pre-digestion. Digestion was performed using the microwave digestion system. After cooling, digested samples were transferred into volumetric flasks and diluted to 25 mL with ultrapure water.

An Inductively Coupled Plasma Optical Emission Spectrometer (ICP-OES) was used for the determination of the following: sodium (Na), potassium (K), calcium (Ca), magnesium (Mg), iron (Fe), manganese (Mn), zinc (Zn), copper (Cu) and sulfur (S). An Inductively Coupled Plasma Mass Spectrometer (ICP-MS) was used for the detection of the following: cobalt (Co) and selenium (Se).

All elemental standard solutions used in this study were obtained from the National Nonferrous Metals and Electronic Materials Analysis and Testing Center, each with a certified concentration of 1000 μg/mL (k = 2). These included:

Potassium (K): GSB-04-1733-2004

Calcium (Ca): GSB-04-1720-2004

Sodium (Na): GSB-04-1738-2004

Magnesium (Mg): GSB-04-1735-2004

Iron (Fe): GSB-04-1726-2004

Manganese (Mn): GSB-04-1736-2004

Copper (Cu): GSB-04-1725-2004

Zinc (Zn): GSB-04-1761-2004

Selenium (Se): GSB-04-1751-2004

Cobalt (Co): GSB-04-1722-2004

Sulfur: GBW(E)085593

Reference values for mineral concentrations were primarily adopted from the published veterinary literature on yaks. The cited references describe the rearing conditions of healthy yak calves in detail, thereby guaranteeing the objectivity and accuracy of the reference values.

### 2.7. Data Analysis

All data were processed using Microsoft Excel and SPSS Statistics 26.0. Descriptive statistics (mean ± standard deviation) were calculated for each element. A Pearson correlation analysis was used to examine the relationships between mineral concentrations and altitude.

The deficiency rate (%) for each element was calculated using the following formula:

Deficiency Rate (%) = (Reference Value − Measured Value)/Reference Value × 100%

## 3. Results

### 3.1. Analysis of Mineral Element Content in Yak Hair and Correlation with Altitude

The data for macroelement contents in yak hair from six regions of Ganzi are presented in Table 2. Reference values were cited from literature [2,3]. The reference values for Na, K, Ca, Mg, and S in hair were 682.51, 66.54, 1989, 285, and 30,000 mg/kg, respectively. The results indicated that Na contents in yak hair from Ganzi and Shiqu were above reference values with no deficiency observed, whereas Na deficiency occurred to varying degrees in Jiulong, Seda, Luhuo, and Yajiang, with deficiency rates of 48.1%, 37.5%, 11.7%, and 2%, respectively. Ca contents in yak hair were above reference values in all regions, with no deficiency detected. Mg content in hair was deficient only in Shiqu, with a deficiency rate as high as 67.0%. S contents were slightly below reference values only in Luhuo and Yajiang, with no deficiency observed in other regions. The data for trace element contents in yak hair from six regions of Ganzi are presented in Table 2. Reference values were cited from the literature [24]: Cu, Fe, Mn, Zn, Co, and Se were 6.42, 391, 15.93, 102.37, 0.76 and 0.49 mg/kg, respectively. The results indicated that Cu contents in yak hair from all six regions were above reference values, with no deficiency observed. Fe contents in yak hair from Luhuo, Seda, and Jiulong were above reference values with no deficiency detected, whereas Fe deficiency occurred to varying degrees in Shiqu, Yajiang, and Ganzi, with deficiency rates of 58.1%, 37.9%, and 34.5%, respectively. Mn content in yak hair from Shiqu was below the reference value, with a deficiency rate of 47.1%, while no Mn deficiency was observed in other regions. No Zn deficiency was detected in any of the six regions. Co deficiency occurred to varying degrees in all six regions, with deficiency rates in Shiqu, Seda, Ganzi, Yajiang, Jiulong, and Luhuo of 89.5%, 78.4%, 78.4%, 73.4%, 57.9%, and 54.0%, respectively. Except for Shiqu, Se deficiency occurred to varying degrees in the remaining five regions, with deficiency rates in Seda, Jiulong, Luhuo, Ganzi, and Yajiang of 74.7%, 63.3%, 48.2%, 44.5%, and 32.7%, respectively.

Correlation analyses between mineral element contents in yak hair and altitude are presented in Table 3. The results demonstrated Mn content was negatively correlated with altitude (r = −0.89, *p* < 0.05), decreasing with increasing altitude.

### 3.2. Analysis of Serum Mineral Element Content in Yaks and Correlation with Altitude

The data for five macroelement contents in yak serum from six regions of Ganzi are presented in Table 4. Reference values were cited from literature [12,25]: Na, K, Ca, Mg, and S were 3000–3500, 90–234, 90, 25, and 1276 mg/L, respectively. The results indicated that Na contents in yak serum from all six regions were above reference values except for Luhuo and Shiqu, which were slightly below reference values, with no deficiency observed in other regions. Ca content in yak serum was below the mean reference value only in Shiqu, with a deficiency rate of 8%, while all other regions were above reference values with no deficiency detected. Mg contents were above reference values only in Seda and Jiulong, whereas varying degrees of deficiency occurred in Luhuo, Shiqu, Ganzi, and Yajiang, with deficiency rates of 39.3%, 32.9%, 9.8%, and 5.0%, respectively. K contents in all six regions were above reference values with no deficiency observed. S deficiency occurred to varying degrees in all six regions, with deficiency rates in Yajiang, Ganzi, Luhuo, Seda, Shiqu, and Jiulong of 41.3%, 37.7%, 36.0%, 30.2%, 25.7%, and 6.8%, respectively. The data for trace element contents in yak serum are presented in Table 4. Reference values for Cu, Fe, Mn, Zn, Co, and Se in serum were 0.6–1.2, 1.1, 0.006, 0.6, 0.4, and 0.07 mg/L, respectively [3,5]. The results indicated that Cu contents in serum were below reference values in all six regions except Jiulong, with varying degrees of deficiency observed; deficiency rates in Seda, Shiqu, Yajiang, Ganzi, and Luhuo were 47.2%, 35%, 31.3%, 20.3%, and 8.0%, respectively. Fe, Mn, Zn, and Co contents in all six regions were above reference values with no deficiency detected. Se deficiency occurred only in Seda and Shiqu, with deficiency rates of 71.4% and 42.8%, respectively.

Correlation analyses between mineral element contents in yak serum and altitude are presented in Table 5. The results demonstrated that Fe content was positively correlated with altitude (r = 0.88, *p* < 0.05), increasing with ascending altitude. Mn content was positively correlated with altitude (r = 0.91, *p* < 0.05), increasing with ascending altitude. Co content was negatively correlated with altitude (r = −0.95, *p* < 0.01), decreasing with ascending altitude.

### 3.3. Analysis of Mineral Element Contents in Forage and Their Correlation with Altitude

The data for mineral element contents in forage from seven regions of Ganzi are presented in Table 6.

Correlation analyses between mineral element contents in forage samples and altitude are presented in Table 7. The results demonstrated Fe content was positively correlated with altitude, increasing with ascending altitude (r = 0.81, *p* < 0.05).

### 3.4. Analysis of Mineral Element Contents in Soil and Their Correlation with Altitude

The data for mineral element contents in soil from seven regions of Ganzi are presented in Table 8.

Correlation analyses between mineral element contents in soil and altitude are presented in Table 9. The results demonstrated Zn content was negatively correlated with altitude, decreasing with ascending altitude (r = −0.80, *p* < 0.05).

### 3.5. Correlation Analysis of Mineral Element Contents in Soil, Forage, Hair, and Serum Across Six Regions

The correlations of mineral element contents among soil, forage, hair, and serum in the Luhuo region are presented in Table 10. The results showed that trace element zinc (Zn) in forage and hair was significantly negatively correlated.

The correlations of mineral element contents among soil, forage, hair, and serum in the Ganzi region are presented in Table 11. The results showed that macroelement sulfur (S) in forage and hair was significantly positively correlated.

The correlations of mineral element contents among soil, forage, hair, and serum in the Yajiang region are presented in Table 12. The results showed that trace element selenium (Se) in forage and serum was significantly negatively correlated.

The correlations of mineral element contents among soil, forage, hair, and serum in the Seda region are presented in Table 13. The results showed that macro-element sodium (Na) in soil and hair was significantly positively correlated, and trace element selenium (Se) in soil and forage was significantly positively correlated.

The correlations of mineral element contents among soil, forage, hair, and serum in the Shiqu region are presented in Table 14. The results showed that trace element cobalt (Co) in forage and serum was significantly positively correlated.

The correlations of mineral element contents among soil, forage, hair, and serum in the Jiulong region are presented in Table 15. The results showed that macro-element sulfur (S) in soil and serum was significantly positively correlated, and trace element cobalt (Co) in forage and hair, as well as iron (Fe) in soil and hair, were significantly negatively correlated.

## 4. Discussion

This study investigated mineral element deficiencies in four types of samples (hair, serum, forage, and soil) across seven regions. Deficiencies were more prevalent in hair and serum, involving Na, Mg, S, Cu, Co, and Se. These findings emphasize the importance of developing targeted mineral supplementation strategies for localized deficiencies to safeguard the health and development of yak calves.

Manganese is closely associated with bone formation and reproductive performance in yaks [3]. Manganese deficiency directly impairs the resistance of yaks to pathogens, leading to issues such as diarrhea [25]. Mn content in hair was negatively correlated with altitude (r = −0.89, *p* < 0.05), with Mn deficiency being more prevalent at higher altitudes. This may be attributed to the fact that higher altitudes are generally characterized by lower temperatures, which weaken soil microbial activity and slow organic matter decomposition, thereby potentially reducing the release of bioavailable manganese (Mn) in the soil and consequently decreasing Mn uptake and accumulation by forage plants. Soil Mn is directly transferred to forage, and as the primary dietary source for yaks, forage Mn content represents the principal determinant of animal Mn intake [12,26]. Therefore, Mn deficiency is essentially absent in lower-altitude regions, whereas Shiqu, as a high-altitude area, exhibits a deficiency that warrants appropriate Mn supplementation.

Sodium is an essential electrolyte involved in numerous physiological functions, including fluid balance and cellular activities. Sodium deficiency can lead to lethargy, anorexia, poor coat condition, and severe neurological symptoms in advanced cases [27]. Our results indicated varying degrees of Na deficiency in hair samples from Jiulong, Seda, Luhuo, and Yajiang, with deficiency rates of 48.1%, 37.5%, 11.7%, and 2%, respectively. The results suggest that the trace element sodium has been chronically deficient in yaks from these four regions. However, serum Na levels in all six regions remained within reference ranges, possibly attributable to recent dietary adjustments. Under natural conditions, soil sodium is released through weathering and leaching processes and subsequently absorbed by plant roots. When soil Na concentrations are elevated, plants tend to accumulate more sodium, which is then ingested by yaks and ultimately reflected in hair. This typical “soil–plant–animal” transfer chain constitutes the fundamental mechanism underlying the positive correlation between soil and hair Na concentrations [28,29]. However, the Na deficiency observed in yak hair from Seda County suggests low soil Na levels in this region, likely attributable to intense soil weathering and strong leaching that result in substantial Na loss, thereby reducing plant-available Na [30].

Calcium (Ca) is one of the most abundant mineral elements in animals, with essential functions in bone and tooth formation, muscle contraction, nerve impulse conduction, and blood coagulation. Hypocalcemia can lead to decreased excitability of systemic muscles, directly increasing the risk of diseases such as ruminal tympany (bloat) and abomasal displacement in cattle [31]. The serum calcium concentrations in yaks from all five regions in Ganzi Prefecture exceeded the normal reference range. We attribute this observation to the following factors. First, August coincides with the peak growing season of alpine forage, during which the herbage is rich in calcium and yaks exhibit high feed intake. Second, intense solar radiation at high altitudes reaches its annual peak during this period, stimulating substantial cutaneous synthesis of vitamin D in yaks, which markedly enhances the intestinal absorption of calcium from the forage. Consequently, the combined effects of high dietary calcium availability and vitamin D-mediated efficient absorption result in serum calcium levels that exceed the normal physiological average.

Magnesium serves as a cofactor for many enzymes involved in various physiological and biochemical reactions, playing critical roles in cardiovascular protection and skeletal health. Mg deficiency in cattle can cause restlessness, tremors, frothing, and convulsions, compromising animal welfare [32]. Our findings revealed that Mg content in hair was deficient only in Shiqu, with a remarkably high deficiency rate of 67.0%. In contrast, serum analysis demonstrated varying degrees of Mg deficiency in Luhuo, Shiqu, Ganzi, and Yajiang, with deficiency rates of 39.3%, 32.9%, 9.8%, and 5.0%, respectively. Both hair and serum samples from Shiqu County showed magnesium deficiency, indicating that yaks in this region experience magnesium depletion both chronically and acutely; therefore, moderate magnesium supplementation is recommended. In contrast, the serum magnesium deficiency observed in the other three regions may be attributed to summer conditions, where a marked increase in the temperature-humidity index directly causes a sharp decline in serum magnesium levels in animals [33]. Furthermore, studies have shown that even when pre-grazing serum magnesium levels appear adequate, exposure to summer pastures or stressful environmental changes (e.g., herd relocation, high temperatures in enclosures) can lead to a rapid decrease in serum magnesium concentration within a short period [34].

Sulfur constitutes an essential component of sulfur-containing amino acids, participating in protein structure, coenzyme and vitamin synthesis, and connective tissue formation [35]. Yaks obtain sulfur primarily through forage consumption, and this sulfur is utilized for the synthesis of sulfur-containing amino acids in vivo, which are subsequently transported to hair follicles to support hair growth [36,37]. An increase in forage sulfur content directly elevates the available sulfur reserves in yaks, ultimately resulting in a concomitant increase in hair sulfur levels [38]. The absence of sulfur deficiency in yak hair samples from the Ganzi region (Table 2) indirectly indicates that forage sulfur content in this region is abundant. However, the sulfur deficiency observed in serum samples from the Ganzi region (Table 4) may be attributed to excessive intake of or exposure to metal ions (e.g., via feed or the environment); sulfur may bind extensively with metal ions such as silver and copper, forming insoluble sulfide deposits in hair or other tissues, thereby reducing circulating free sulfur and resulting in decreased serum sulfur levels [39]. In the Jiulong region, sulfur exhibited a positive correlation between soil and serum. This can be explained by the fact that forage plants absorb sulfur from the soil and convert it into sulfur-containing amino acids. During summer, when forage growth is vigorous, yaks consume substantial quantities of herbage, and serum sulfur levels would be expected to rise following digestion and absorption [40]. However, the slightly subnormal serum sulfur levels observed in the Jiulong region may be attributed to excessive intake of or exposure to metal ions (e.g., via feed or the environment), a situation similar to that observed in the Ganzi region.

Copper functions as a critical component of the active centers of numerous enzymes, extensively participating in energy metabolism, antioxidant functions, and elastin and collagen production. Cu deficiency predisposes cattle to cardiovascular diseases, compromised immune responses, growth retardation and diarrhea in calves, reduced fertility and sperm quality in bulls, and impaired steroid hormone synthesis in ovarian granulosa cells [41]. Our results demonstrated that hair Cu contents were normal across all six regions, whereas serum Cu deficiency occurred to varying degrees in all six regions. Serum copper primarily reflects recent (hours to days) dietary copper intake and the dynamic equilibrium of systemic copper metabolism, whereas hair copper represents long-term copper accumulation over weeks to months [42]. The copper deficiency observed in serum from five regions (excluding Jiulong) may be attributable to antagonistic effects caused by high molybdenum or iron contents in soil and forage, resulting in relatively low bioavailability of copper in yak milk or dietary rations [13,43]. As a terminal tissue, hair exhibits slower copper deposition kinetics and possesses inherent buffering capacity; consequently, short-term insufficient copper intake may not yet significantly alter the total copper accumulation in hair, thereby leading to apparently normal hair copper levels.

Cobalt serves as the core element of vitamin B12, participating in erythrocyte maturation and thereby influencing hematopoietic function. It is also involved in energy metabolism and protein synthesis [44]. Co deficiency can result in anorexia, rough and dull hair coat, anemia, developmental retardation, and emaciation [45]. In our study, varying degrees of Co deficiency were detected in hair samples from all six regions, whereas serum Co contents in all six regions exceeded reference values. Cobalt exhibits a significant biological transmission relationship among soil, forage, and animal blood [46]. Previous studies have demonstrated that lower pH values (acidic conditions) increase the solubility and bioavailability of metal ions such as cobalt, facilitating their absorption by forage roots, whereas higher pH values (alkaline conditions) promote metal ion precipitation or adsorption, thereby reducing their availability [47]. During summer, when forage is abundant, forage serves as the primary dietary source of cobalt for ruminants. Following consumption by yaks, cobalt is utilized by rumen microorganisms for vitamin B_12_ synthesis, directly confirming the biological transmission efficiency of cobalt through the food chain [48,49]. The positive correlation between serum and forage cobalt concentrations in the Shiqu region, together with the absence of serum cobalt deficiency in the Jiulong region, provides corroborating evidence for this biological transmission pathway. Additionally, the subnormal cobalt levels detected in yak hair from Shiqu and Jiulong may be attributed to the cumulative effect of sustained low cobalt nutritional status over the preceding months, particularly during the winter and spring seasons.

The primary physiological function of selenium involves serving as the core component of glutathione peroxidase (GPX), catalyzing the decomposition of peroxides and synergistically acting with vitamin E as antioxidants, playing crucial roles in protecting cellular structures and protein synthesis [50]. Previous studies have indicated that Se deficiency primarily affects normal growth and development in calves, impedes fat and vitamin E metabolism and utilization, and predominantly causes necrosis of cardiac and skeletal muscle, with affected muscle regions losing their original color and presenting pallor (white muscle disease) [51]. Forage plants primarily absorb selenium from the soil through their roots, mainly in the forms of selenate and selenite. Selenate is an inorganic form of selenium with relatively low bioavailability [52]. Conversely, forage plants can convert selenium into organic forms through their own metabolic pathways, which represents the primary absorption mechanism in animals [53]. During summer, when forage supply is abundant, yaks may maintain metabolic homeostasis by enhancing selenium excretion (e.g., via feces and urine) or transferring selenium into milk (in lactating cows), thereby preventing a significant increase in serum selenium and preserving steady-state levels [54]. Additionally, another possible explanation is that previous studies have demonstrated that under alkaline and oxidizing soil conditions, selenium exists predominantly in the form of selenate, which is readily absorbed by plants. The soil characteristics in the Yajiang region may result in relatively low selenium bioavailability; although total selenium content may not be deficient, a greater proportion of selenium within plants exists as inorganic forms with lower bioavailability [55]. This may account for the significant negative correlation between forage and serum selenium observed in the Yajiang region. Previous studies have demonstrated that soil selenium content is a critical determinant of forage selenium nutritional status, and total soil selenium content is typically significantly positively correlated with forage selenium levels, which is consistent with the correlation analysis results between soil and forage selenium in Seda [52,56]. However, the observed selenium deficiency in yak hair and serum in the Seda region may be attributable to the influence of soil pH and other factors on forage selenium speciation; a greater proportion of selenium within the forage may exist as inorganic forms, resulting in insufficient bioavailability for the animals [55].

Zinc is an essential cofactor for numerous enzymes and participates in the synthesis, storage, and release of insulin, growth hormone, and other biomolecules, playing a critical role in bone development, tissue regeneration, and repair processes [57,58,59]. Zinc deficiency can lead to multiple disorders, including growth retardation, compromised immunity, and cardiovascular diseases [60,61]. We found no evidence of zinc (Zn) deficiency in yak hair or serum samples from Ganzi Prefecture. However, soil Zn exhibited a significant negative correlation with altitude, decreasing with increasing elevation. This pattern may be explained by the fact that trace element concentrations in remote high-altitude areas are typically low, primarily originating from atmospheric deposition rather than local primary release. Soil Zn content at high elevations reflects the combined effects of natural weathering processes and minor contributions from long-distance atmospheric transport, while lacking localized anthropogenic enrichment sources [62]. Furthermore, microbial community structure in high-altitude regions is significantly influenced by elevation; changes in soil microbial activity and composition may affect Zn speciation transformation and bioavailability [63]. In Luhuo region, zinc content in animals is regulated by multiple physiological processes [64]. When forage zinc levels increase, animals may activate homeostatic regulatory mechanisms, such as enhancing excretion or reducing absorption efficiency, to maintain internal environmental stability. This may lead to increased zinc excretion in feces, thereby making tissue zinc concentrations relatively stable or even decreased [65].

This study has several limitations. First, the relatively small sample size (42 animals) and the restricted geographical scope (six regions within Ganzi Prefecture) may limit the generalizability of our findings. Second, hair and blood samples from yaks were collected during a single sampling campaign within one year, potentially compromising temporal representativeness. Third, the number and spatial coverage of soil and forage sampling sites were limited. Future research should address these gaps by expanding the sample size, conducting multi-time-point or multi-year sampling, and increasing both the number and spatial extent of soil and forage sampling sites.

## 5. Conclusions

This study aimed to investigate the mineral element status of healthy yak calves in Ganzi Prefecture, as well as the mineral element contents in soil and forage. It elucidated the deficiency patterns and spatial distribution characteristics of mineral elements across different altitudinal gradients and explored the levels and interrelationships of mineral elements within the “soil–forage–animal” ecosystem. The results revealed that deficiencies of Na, Mg, Co, Se, and Cu were prevalent. Complex correlations among mineral elements in soil, forage, hair, and serum were observed across different regions, and these correlations varied due to factors such as regional differences in soil and forage mineral element contents and livestock activity. These findings provide a scientific basis for developing targeted mineral supplementation strategies and ecological management practices, thereby helping to ensure yak health and promote the sustainable development of livestock farming in high-altitude pastoral areas.

## Figures and Tables

**Table 1:** Grazing area, botanical composition, and dominant forage species in seven sampling regions.

County	Daocheng (3750 m)	Luhuo (3200 m)	Ganzi (3410 m)	Yajiang (3600 m)	Seda (3900 m)	Shiqu (4200 m)	Jiulong (4100 m)
grazing area (thousand hectares)	88.00	276.67	461.33	335.33	572.10	1920.13	306.67
plant species	*Ranunculus japonicu*, *Gentiana macrophylla*, *Elymus nutans*	*Pteridium aquilinum*, *Elymus nutans*, *Hippophae rhamnoides*	*Kobresia uncinoides*, *Triglochin palustris*, *Rheum palmatum*	*Kobresia pygmaea*, *Potentilla anserina*, *Caltha scaposa*	*Kobresia pygmaea*, *Potentilla chinensis*, *Oxytropis ochrocephala*	*Elymus nutans, Rhodiola crenulata*, *Polygonum viviparum*	*Elymus sibiricus*, *Rhododendron lapponicum*, *Rheum palmatum*
forage species	*Elymus nutans*	*Elymus nutans*	*Kobresia uncinoides*	*Kobresia pygmaea*.	*Kobresia pygmaea*	*Elymus nutans*	*Elymus sibiricus*

**Table 2:** Comparison of mineral element contents (mg/kg, mean ± SD) in yak hair samples from six regions with reference values.

Element	Reference	Luhuo (3200 m)	Ganzi (3410 m)	Yajiang (3600 m)	Seda (3900 m)	Shiqu (4200 m)	Jiulong (4100 m)
Na (mg/kg)	682.51	602.22 ± 113.62	731.61 ± 139.89	671.60 ± 197.65	416.62 ± 101.83	1635.49 ± 358.97	353.62 ± 193.48
Ca (mg/kg)	1989	3828.79 ± 710.54	3056.22 ± 580.43	3856.71 ± 911.78	3181.87 ± 222.09	2421.04 ± 654.40	2242.78 ± 493.01
Mg (mg/kg)	285	701.96 ± 146.95	757.99 ± 96.04	737.25 ± 205.49	582.91 ± 156.80	94.23 ± 253.65	474.91 ± 47.37
S (mg/kg)	30,000	29,936.97 ± 1461.09	33,895.63 ± 1452.71	29,048.92 ± 874.07	31,839.20 ± 2565.03	34,097.13 ± 1729.81	36,283.13 ± 2342.43
Cu (mg/kg)	6.42	9.80 ± 0.43	10.93 ± 1.51	8.75 ± 0.72	8.72 ± 1.02	9.16 ± 0.85	6.43 ± 0.98
Fe (mg/kg)	391	598.76 ± 151.25	256.16 ± 57.30	243.22 ± 62.03	515.67 ± 264.45	163.65 ± 63.68	565.69 ± 119.35
Mn (mg/kg)	15.93	63.18 ± 10.69	43.16 ± 9.01	59.96 ± 16.82	39.02 ± 11.88	8.43 ± 5.25	24.06 ± 7.41
Zn (mg/kg)	102.37	108.20 ± 3.42	126.00 ± 4.12	171.81 ± 11.71	111.37 ± 13.05	178.33 ± 58.25	105.29 ± 6.97
Co (mg/kg)	0.76	0.35 ± 0.08	0.16 ± 0.04	0.20 ± 0.05	0.16 ± 0.04	0.08 ± 0.03	0.32 ± 0.16
Se (mg/kg)	0.49	0.25 ± 0.02	0.27 ± 0.01	0.33 ± 0.02	0.12 ± 0.02	0.49 ± 0.14	0.18 ± 0.01

**Table 3:** Correlation coefficients (r) and *p*-values between mineral element contents (mg/kg, mean ± SD) in yak hair samples from six regions and altitude (* *p* < 0.05).

Element	Correlation Coefficient (r)	*p*-Value	Significance	Correlation Interpretation
Na	0.33	0.51	Not significant	Not significant correlation
Ca	−0.81	0.05	Not significant	Not significant correlation
Mg	−0.60	0.21	Not significant	Not significant correlation
S	0.62	0.19	Not significant	Not significant correlation
Cu	−0.65	0.16	Not significant	Not significant correlation
Fe	−0.18	0.73	Not significant	Not significant correlation
Mn	−0.89	<0.05	*	Significant negative correlation; content decreases with increasing altitude
Zn	0.27	0.61	Not significant	Not significant correlation
Co	−0.43	0.40	Not significant	Not significant correlation
Se	0.18	0.74	Not significant	Not significant correlation

**Table 4:** Comparison of mineral element contents (mg/L or μg/L, mean ± SD) in yak serum samples from six regions with reference values.

Element	Reference	Luhuo (3200 m)	Ganzi (3410 m)	Yajiang (3600 m)	Seda (3900 m)	Shiqu (4200 m)	Jiulong (4100 m)
Na (mg/L)	3000–3500	2896.52 ± 238.15	3070.97 ± 159.52	3124.13 ± 346.99	3159.57 ± 124.44	2900.11 ± 69.55	3212.90 ± 77.89
Ca (mg/L)	90	93.65 ± 9.87	101.21 ± 2.42	94.30 ± 8.13	300.36 ± 10.05	82.63 ± 5.94	237.90 ± 18.84
Mg (mg/L)	25	15.17 ± 1.95	22.55 ± 1.69	23.73 ± 5.51	38.01 ± 1.51	16.78 ± 1.75	29.90 ± 2.36
K (mg/L)	97–234	131.20 ± 12.36	167.31 ± 12.51	193.09 ± 10.39	211.65 ± 20.44	176.84 ± 16.83	173.98 ± 20.71
S (mg/L)	1276	816.45 ± 63.19	795.16 ± 47.09	749.61 ± 68.17	891.63 ± 84.77	947.83 ± 86.74	1189.85 ± 41.14
Cu (mg/L)	0.6–1.2	0.55 ± 0.09	0.47 ± 0.05	0.41 ± 0.05	0.31 ± 0.08	0.39 ± 0.06	0.81 ± 0.09
Fe (mg/L)	1.1	2.46 ± 0.27	2.90 ± 0.17	1.79 ± 0.29	8.72 ± 0.85	8.06 ± 1.27	9.39 ± 0.82
Mn (mg/L)	0.006	0.05 ± 0.01	0.04 ± 0.01	0.048 ± 0.08	0.29 ± 0.02	0.29 ± 0.09	0.31 ± 0.05
Zn (mg/L)	0.6	0.88 ± 0.10	0.62 ± 0.06	0.67 ± 0.09	1.12 ± 0.05	1.06 ± 0.36	1.42 ± 0.13
Co (ug/L)	0.4	9.76 ± 1.48	6.33 ± 1.86	6.01 ± 0.76	1.81 ± 0.31	1.61 ± 0.60	2.51 ± 0.91
Se (mg/L)	0.07	0.13 ± 0.02	0.07 ± 0.01	0.12 ± 0.020	0.02 ± 0.05	0.04 ± 0.01	0.12 ± 0.04

**Table 5:** Correlation coefficients (r) and *p*-values between mineral element contents (mg/L or μg/L, mean ± SD) in yak serum samples from six regions and altitude (* *p* < 0.05; ** *p* < 0.01).

Element	Correlation Coefficient (r)	*p*-Value	Significance	Correlation Interpretation
Na	0.28	0.55	Not significant	Not significant correlation
Ca	0.43	0.40	Not significant	Not significant correlation
Mg	0.38	0.47	Not significant	Not significant correlation
K	0.56	0.25	Not significant	Not significant correlation
S	0.71	0.11	Not significant	Not significant correlation
Cu	0.07	0.90	Not significant	Not significant correlation
Fe	0.88	<0.05	*	Significant positive correlation; content increases with increasing altitude
Mn	0.91	<0.05	*	Significant positive correlation; content increases with increasing altitude
Zn	0.72	0.11	Not significant	Not significant correlation
Co	−0.95	<0.01	**	Significant negative correlation; content decreases with increasing altitude
Se	0.09	0.87	Not significant	Not significant correlation

**Table 6:** Mineral element contents (mg/kg, mean ± SD) in forage samples from seven regions.

Element	Daocheng (3750 m)	Luhuo (3200 m)	Ganzi (3410 m)	Yajiang (3600 m)	Seda (3900 m)	Shiqu (4200 m)	Jiulong (4100 m)
Na (mg/kg)	108.13 ± 25.45	44.54 ± 9.66	78.08 ± 19.38	67.92 ± 27.50	101.03 ± 4.74	207.98 ± 53.69	400.13 ± 140.39
Ca (mg/kg)	11,263.65 ± 1686.73	7660.35 ± 2348.01	5971.35 ± 281.3	7752.04 ± 1814.11	6397.15 ± 209.47	7157.33 ± 734.13	12,547.42 ± 2885.67
Mg (mg/kg)	3603.14 ± 537.61	1881.06 ± 519.68	1647.97 ± 559.56	2895.76 ± 921.34	1550.68 ± 124.15	1671.80 ± 271.34	2613.42 ± 388.39
K (mg/kg)	44,014.57 ± 561.60	19,211.30 ± 588.19	28,770.56 ± 103 4.55	31,099.33 ± 3757.37	11,800.62 ± 500.35	19,071.26 ± 723.68	7161.31 ± 1583.67
S (mg/kg)	3832.73 ± 359.78	1407.01 ± 354.87	1884.90 ± 525.18	2752.53 ± 297.65	1435.77 ± 374.33	2015.96 ± 527.57	1322.55 ± 158.02
Cu (mg/kg)	15.42 ± 1.72	7.83 ± 1.55	6.93 ± 1.75	12.63 ± 2.33	26.70 ± 10.48	7.23 ± 0.56	26.14 ± 2.99
Fe (mg/kg)	662.30 ± 210.87	417.21 ± 83.92	688.55 ± 317.98	222.61 ± 93.70	2138.74 ± 975.34	1813.25 ± 884.91	2622.35 ± 462.81
Mn (mg/kg)	168.60 ± 41.25	141.46 ± 38.95	176.76 ± 90.79	181.12 ± 107.43	145.94 ± 28.77	91.05 ± 12.14	278.05 ± 84.07
Zn (mg/kg)	94.56 ± 14.94	34.84 ± 3.52	31.38 ± 8.44	54.34 ± 15.29	33.10 ± 3.24	35.82 ± 7.65	38.10 ± 5.14
Co (mg/kg)	0.29 ± 0.08	0.33 ± 0.03	0.39 ± 0.18	0.51 ± 0.31	0.54 ± 0.26	0.61 ± 0.27	1.23 ± 0.16
Se (mg/kg)	0.06 ± 0.01	0.05 ± 0.01	0.13 ± 0.03	0.11 ± 0.05	0.06 ± 0.03	0.12 ± 0.04	0.12 ± 0.03

**Table 7:** Correlation coefficients (r) and *p*-values between mineral element contents (mg/kg, mean ± SD) in forage samples from seven regions and altitude (* *p* < 0.05;).

Element	Correlation Coefficient (r)	*p*-Value	Significance	Correlation Interpretation
Na	0.74	0.06	Not significant	Not significant correlation
Ca	0.35	0.44	Not significant	Not significant correlation
Mg	0.04	0.93	Not significant	Not significant correlation
K	−0.27	0.56	Not significant	Not significant correlation
S	−0.03	0.95	Not significant	Not significant correlation
Cu	0.49	0.26	Not significant	Not significant correlation
Fe	0.81	<0.05	*	Significant positive correlation; content increases with increasing altitude
Mn	0.08	0.86	Not significant	Not significant correlation
Zn	0.01	0.98	Not significant	Not significant correlation
Co	0.64	0.12	Not significant	Not significant correlation
Se	0.34	0.46	Not significant	Not significant correlation

**Table 8:** Trace element contents (mg/kg, mean ± SD) in soil samples from seven regions.

Element	Daocheng (3750 m)	Luhuo (3200 m)	Ganzi (3410 m)	Yajiang (3600 m)	Seda (3900 m)	Shiqu (4200 m)	Jiulong (4100 m)
Na (mg/kg)	4462.71 ± 656.44	8260.86 ± 2107.51	8772.20 ± 420.90	4229.29 ± 96.18	11,101.86 ± 1925.59	10,410.86 ± 531.40	9044.00 ± 295.72
Ca (mg/kg)	2664.25 ± 259.78	4913.86 ± 860.09	5445.10 ± 1165.88	2224.29 ± 391.56	5997.17 ± 748.91	4287.86 ± 1252.11	5119.01 ± 1790.52
Mg (mg/kg)	5117.43 ± 786.73	7164.43 ± 792.73	7914.29 ± 217.80	11167.71 ± 203.30	7354.86 ± 392.93	8926.00 ± 765.57	8932.00 ± 654.71
K (mg/kg)	19257.14 ± 327.10	19483.71 ± 137.67	20577.14 ± 100.64	16083.85 ± 426.75	21800.00 ± 226.41	17514.28 ± 959.91	12914.29 ± 681.73
S (mg/kg)	531.55 ± 98.13	681.32 ± 97.12	434.80 ± 62.43	641.33 ± 20.35	682.82 ± 63.21	455.72 ± 57.44	430.05 ± 44.42
Cu (mg/kg)	18.01 ± 2.71	23.83 ± 1.70	24.77 ± 4.12	20.27 ± 0.50	23.23 ± 3.26	19.14 ± 1.05	25.40 ± 1.52
Fe (mg/kg)	29,887.57 ± 7216.22	31,528.43 ± 1982.77	33,559.14 ± 1056.40	38,192.86 ± 844.30	33,324.43 ± 1020.44	27,860.29 ± 1506.07	34,083.86 ± 985.89
Mn (mg/kg)	730.40 ± 196.48	809.74 ± 73.53	852.86 ± 51.82	629.80 ± 14.70	717.140 ± 77.33	658.00 ± 34.46	549.57 ± 106.64
Zn (mg/kg)	99.56 ± 18.30	103.47 ± 21.82	87.31 ± 8.86	100.27 ± 2.20	85.07 ± 5.46	65.57 ± 6.96	80.14 ± 3.41
Co (mg/kg)	7.86 ± 1.33	12.42 ± 1.73	14.49 ± 0.53	11.60 ± 0.31	12.56 ± 0.82	10.23 ± 0.49	13.90 ± 0.62
Se (mg/kg)	0.13 ± 0.06	0.13 ± 0.02	0.12 ± 0.03	0.23 ± 0.03	0.23 ± 0.01	0.20 ± 0.03	0.67 ± 0.25

**Table 9:** Correlation coefficients (r) and *p*-values between trace element contents (mg/kg, mean ± SD) in soil samples from seven regions and altitude (* *p* < 0.05).

Element	Correlation Coefficient (r)	*p*-Value	Significance	Correlation Interpretation
Na	0.35	0.44	Not significant	Not significant correlation
Ca	0.74	0.06	Not significant	Not significant correlation
Mg	0.43	0.33	Not significant	Not significant correlation
K	−0.43	0.34	Not significant	Not significant correlation
S	−0.44	0.32	Not significant	Not significant correlation
Cu	−0.29	0.53	Not significant	Not significant correlation
Fe	−0.29	0.53	Not significant	Not significant correlation
Mn	−0.75	0.05	Not significant	Not significant correlation
Zn	−0.80	<0.05	*	Significant negative correlation; content decreases with increasing altitude
Co	−0.22	0.64	Not significant	Not significant correlation
Se	0.56	0.20	Not significant	Not significant correlation

**Table 10:** Correlation coefficients (r) of mineral element contents among soil, forage, yak hair and serum in Luhuo Region (* *p* < 0.05).

Item	Na	Ca	Mg	S	Cu	Fe	Mn	Zn	Co	Se
soil–forage	0.07	0.73	0.11	0.52	−0.57	−0.38	0.33	0.31	−0.06	−0.43
soil–serum	0.25	0.11	0.39	−0.24	0.03	0.57	0.02	−0.74	0.28	0.27
soil–hair	0.07	0.79	0.51	0.99	0.09	0.45	−0.39	−0.31	0.16	0.04
forage–serum	0.18	−0.41	−0.52	−0.02	0.16	0.06	−0.09	−0.09	−0.24	0.51
forage–hair	0.51	−0.07	−0.59	−0.04	−0.64	−0.07	−0.33	−0.79 *	−0.51	0.05

**Table 11:** Correlation coefficients (r) of mineral element contents among soil, forage, yak hair and serum in Ganzi Region (* *p* < 0.05).

Item	Na	Ca	Mg	S	Cu	Fe	Mn	Zn	Co	Se
soil–forage	−0.38	0.56	−0.49	0.24	−0.08	−0.18	0.38	−0.16	0.21	−0.36
soil–serum	−0.18	−0.25	−0.39	−0.50	0.09	−0.99	0.55	−0.34	0.63	−0.42
soil–hair	−0.71	0.27	0.21	−0.24	0.04	−0.09	−0.11	0.69	−0.26	−0.09
forage–serum	−0.19	−0.60	0.49	−0.21	−0.27	0.70	0.19	−0.03	−0.10	−0.07
forage–hair	−0.11	−0.08	−0.68	0.86 *	−0.31	−0.51	0.17	−0.41	0.07	0.48

**Table 12:** Correlation coefficients (r) of mineral element contents among soil, forage, yak hair and serum in Yajiang Region (* *p* < 0.05).

Item	Na	Ca	Mg	S	Cu	Fe	Mn	Zn	Co	Se
soil–forage	0.17	0.04	−0.32	0.47	−0.26	−0.48	−0.04	−0.23	−0.38	0.39
soil–serum	−0.28	0.34	0.03	0.21	0.33	0.34	−0.24	0.01	0.38	−0.40
soil–hair	0.16	0.17	0.39	−0.08	0.57	−0.56	0.04	−0.14	−0.42	−0.19
forage–serum	0.22	0.31	0.11	0.10	−0.24	0.19	0.09	−0.54	0.27	−0.84 *
forage–hair	−0.29	−0.35	−0.46	0.17	−0.40	0.09	−0.28	0.75	0.32	−0.47

**Table 13:** Correlation coefficients (r) of mineral element contents among soil, forage, yak hair and serum in Seda Region (* *p* < 0.05).

Item	Na	Ca	Mg	S	Cu	Fe	Mn	Zn	Co	Se
soil–forage	0.46	0.58	−0.11	0.12	−0.28	0.39	−0.17	0.36	0.21	0.77 *
soil–serum	0.24	−0.08	−0.45	0.14	−0.50	0.34	0.28	0.27	−0.43	0.58
soil–hair	0.84 *	0.35	0.13	0.43	−0.61	0.25	0.11	0.36	−0.01	−0.48
forage–serum	0.39	0.44	0.08	−0.49	−0.13	0.30	0.32	0.35	0.06	0.04
forage–hair	0.17	−0.12	0.17	0.34	−0.39	0.33	0.22	−0.11	−0.23	−0.45

**Table 14:** Correlation coefficients (r) of mineral element contents among soil, forage, yak hair and serum in Shiqu Region (* *p* < 0.05).

Item	Na	Ca	Mg	S	Cu	Fe	Mn	Zn	Co	Se
soil–forage	0.21	0.57	−0.54	0.19	−0.20	0.64	0.24	0.37	0.35	−0.15
soil–serum	−0.38	0.21	−0.02	0.19	−0.14	−0.44	−0.01	−0.35	0.42	0.26
soil–hair	−0.56	0.40	0.54	−0.04	−0.30	−0.61	0.090	−0.30	−0.30	−0.38
forage–serum	0.55	0.23	0.62	0.21	0.48	−0.50	−0.28	−0.08	0.67 *	−0.36
forage–hair	0.27	0.31	−0.10	0.62	0.75	−0.32	−0.21	−0.35	−0.22	0.33

**Table 15:** Correlation coefficients (r) of mineral element contents among soil, forage, yak hair and serum in Jiulong Region (* *p* < 0.05).

Item	Na	Ca	Mg	S	Cu	Fe	Mn	Zn	Co	Se
soil–forage	−0.22	0.21	−0.02	0.48	0.07	0.10	0.53	0.58	0.65	0.35
soil–serum	−0.14	0.24	−0.46	0.83 *	0.06	0.28	−0.07	0.46	−0.13	0.40
soil–hair	0.11	0.14	0.44	0.09	−0.70	−0.93	0.43	0.10	−0.39	0.01
forage–serum	0.06	0.15	0.61	0.17	−0.09	−0.34	0.75	0.50	−0.03	−0.07
forage–hair	−0.24	0.03	−0.43	0.31	−0.16	−0.23	−0.41	−0.41	0.88 *	−0.46

## Data Availability

The data that support the findings of this study are available from the corresponding author upon reasonable request.
